# Adrenal cavernous hemangioma: A rare cause of chronic lumbar pain - a case report

**DOI:** 10.1016/j.ijscr.2024.109936

**Published:** 2024-06-22

**Authors:** Ktari Kamel, Jelassi Mohamed Amine, Wadii Hamdouni, Seifeddine Ben Hammouda, Fedia Boubaker, Jamel Saad

**Affiliations:** a Department Of Urology, Fattouma Bourguiba Hospital, Monastir, Tunisia; b Departement of Anatompathology, Fattouma Bourguiba Hospital, Monastir, Tunisia; c Departement Of Endocrinology, Taher Sfar Hospital, Mahdia, Tunisia; d Departement Of radiology, Fattouma Bourguiba Hospital, Monastir, Tunisia

**Keywords:** Adrenal gland, Neoplasm, Tumor, Hemangioma, Lumbar pain, Case report

## Abstract

**Introduction and importance:**

Adrenal Cavernous Hemangioma is an extremely rare histological type of adrenal tumors, typically asymptomatic and occasionally revealed by a symptom or complication. Here, we report an atypical symptomatic case to enrich the limited international case series.

**Case presentation:**

We present the case of an 80-year-old woman who underwent laparoscopic left adrenalectomy for a painful and potentially malignant left adrenal neoplasm, leading to the discovery of a five-centimeter adrenal cavernous hemangioma. The post-operative course was uneventful. The postoperative course was uneventful, and the chronic lumbar pain described initially vanished at the six-month follow-up.

**Clinical discussion:**

Adrenal cavernous hemangioma is typically silent and incidentally discovered on cross-sectional imaging. Symptomatic or complicated forms are extremely rare. Clinical, biological, radiological and histology assessment are crucial for management. Therapeutic decisions depend on the malignancy probability and the functional nature of the adrenal neoplasm, considering surgery versus conservative approaches. Patient's point-of-view and background are also determining factors in the decision-making process. Mini-invasive adrenalectomy is superior to open approach, when feasible and safe.

**Conclusion:**

Adrenal cavernous hemangioma is a rare benign vascular tumor often discovered on adrenalectomy specimen. This case illustrates a rare cause of chronic lumbar pain. It also underscores the importance of a multidisciplinary medical decision for this kind of tumors.

## Highlights

• Adrenal Cavernous Hemangioma is a rare benign vascular tumor.
• It is usually asymptomatic, occasionally revealed by a symptom or a complication.
• The preoperative diagnosis is very challenging.
• The Therapeutic decisions are based on the malignancy probability, functional nature of the adrenal neoplasm, and complications, balancing surgery versus a conservative approach.

## Abbreviations

(AH): Adrenal Hemangioma
(CD): Clusters of Differentiation
(CT): Computed-Tomography
(DGC): Deterioration of general condition
(HE): Hematoxylin and Eosin
(HU): Hounsfield Units
(MRI): Magnetic Resonance Imaging
(PET): Positron Emission Tomography
(VAS): Visual Analog Scale

## 1 Introduction

With advancements in cross-sectional imaging techniques, the preoperative diagnosis and characterization of adrenal neoplasms have become less challenging adrenal neoplasms are most often incidentally discovered, known as incidentalomas [1]. Less frequently, symptoms such as chronic pain, abdominal compression syndrome, internal bleeding and deterioration of general condition (DGC) may lead to the diagnosis of adrenal neoplasms [[2], [3], [4], [5]]. The challenge is to rule out malignancy (adrenocortical carcinoma, malignant pheochromocytoma, metastatic lesion…) and assess the secretory nature of adrenal neoplasm (catecholamines, aldosterone, cortisol or adrenal androgens). Clinical, biological and radiological features provide valuable information for decision-making process. A multidisciplinary approach, including surgeons, endocrinologists, radiologists and nuclear physicians is essential for deciding between surgical and medical management of adrenal tumors [6].

Adrenal Hemangioma (AH) is a rare, benign vascular tumor, usually asymptomatic [[2], [3], [4],^7^]. Fewer than seventy cases have been reported since 1955. Few cases of symptomatic or complicated AH are found in scientific literature. We report a rare symptomatic case of Adrenal Cavernous Hemangioma in line with SCARE criteria [8] to discuss its features, complications, and management, thereby enriching the scientific literature.

## 2 Presentation of the case

**Fig. 1:**
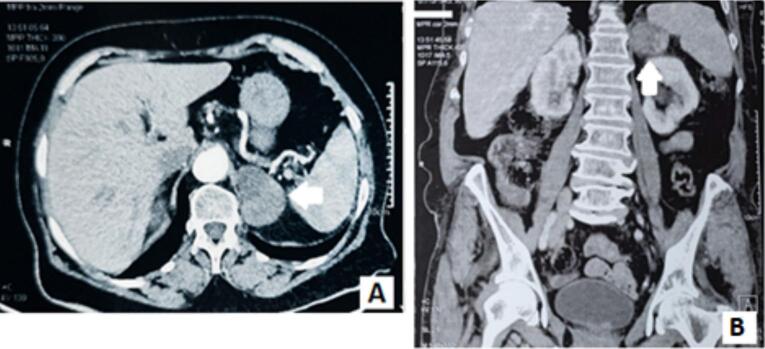
Enhanced abdominal CT-Scan showing a left suprarenal mass. Axial section (A) and frontal reconstruction (B) of the left adrenal mass at arterial and portal phase: showing heterogeneous enhancing of the lesion with low wash-out rate.

An 80-year-old woman, with medical history of well- controlled hypertension, dyslipidemia and gout, presented of chronic left lumbar pain of four months' duration. The pain was moderate (VAS = 4), continuous and partially relieved by paracetamol-codeine association. Anamnesis didn't reveal any other associated symptoms. Physical examination revealed mild lumbar tenderness and a good general condition. Abdominal ultrasound showed a well-demarcated solid lesion of the left adrenal gland, measuring 4 cm in its greater axis. Enhanced abdominal CT-scan revealed a possibly malignant left adrenal mass (49 × 34 mm) with a 22-HU spontaneous density and a 33 % relative wash-out rate (Fig. 1). These findings were in favor of a possibly malignant left adrenal mass. Thoracic CT-scan was normal.

Biological assessment of the adrenal neoplasm was strictly unremarkable: Reviewer 2 and 3

- Normal fasting blood sugar = 5 mmol/L and normal serum potassium = 3.8 mmol/L
- Normal 8 h serum cortisol level = 4.2 μg/dL and positive dexamethasone suppression test: cortisol level after dexamethasone suppression = 1.1 μg/L
- Normal aldosterone/renine ratio = 48 (< 64)
- Normal urinary fractioned metanephrine rates = 81 μg/24 h

Thoracic CT-scan was normal. Further investigation of the mass, using MRI and PET scan, was judged futile.

After a multidisciplinary discussion, laparoscopic left adrenalectomy was performed following the patient's informed consent. The benefit-risk balance of surgical intervention was judged favorable, given patient's good general condition and lack of severe comorbidities.

The woman underwent laparoscopic trans-peritoneal removal of left adrenal gland one month later. We relied on progressive dissection and minimal manipulation of the adrenal gland to minimize the risk of intraoperative bleeding, which was estimated at 100 mL. No adrenal adhesions were found. Total operative time was estimated at 90 min. A Salem probe was kept in the adrenalectomy chamber and removed two days later.

**Fig. 2:**
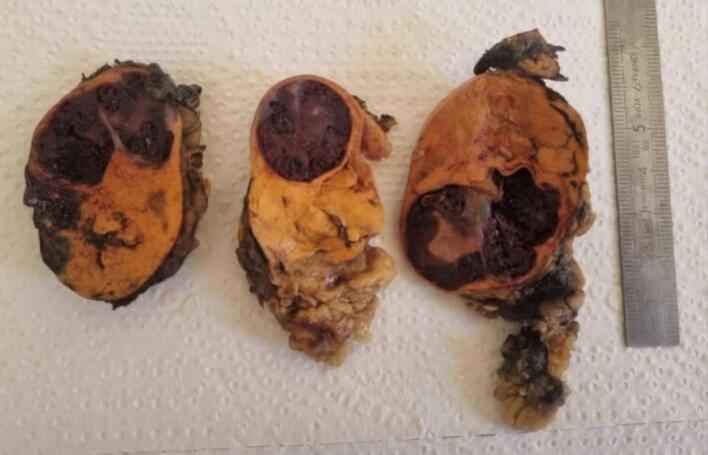
Left adrenal gland with a hemorrhagic solid lesion corresponding to adrenal hemangioma.

**Fig. 3:**
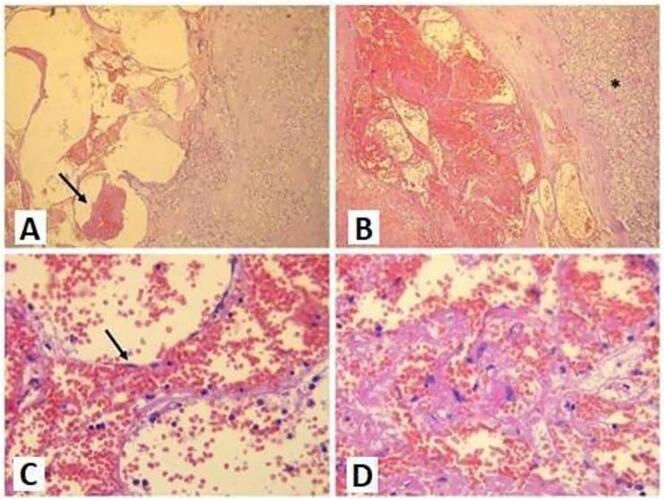
Microscopic study images of an adrenal cavernous hemangioma. (A, B: HE staining ×4): Dilated and tightly packed vessels filled with clots and sometimes thrombi can be seen (A, Black Arrow). These vessels are well demarcated from the adjacent adrenal parenchyma (B, Black Asterisk) and divided by thin fibrous septa. (C, D: HE staining ×40): Vessels are lined by a single layer of regular endothelial cells (C, Black Arrow).

The patient was discharged two days after the surgery. No post-operative complications have been noticed yet. Gross pathological examination of the adrenal specimen showed a 52-g adrenal gland, surrounded by fat tissue, measuring 5.5 * 3.5 * 3 cm, with a 4-cm hemorrhagic lesion (Fig. 2). Histological study of the adrenal specimen concluded to an adrenal cavernous hemangioma (Fig. 3), which is a benign adrenal tumor. A rigorous and regular clinical follow-up of the patient was decided. Chronic lumbar pain - which was the chief complaint-, has completely vanished at sixth month follow-up. That reinforced the assumption of a causality link between the mass and the lumbar pain.

## 3 Discussion

Hemangiomas are rare tumors [9] arising from the endothelial lining of blood vessels. It is thought that embryonic remnants, made of abnormally developed angioblastic cells, are responsible for their genesis. Skin, liver and brain locations are the most frequent [10,11]. Adrenal location is not common [10,11]. Adrenal Hemangiomas are usually benign, non-invasive and non- secretory tumors. Adrenal hemangiomas have a tan-brown and hemorrhagic cut surface.

Calcifications can be seen in AH [12].

Adrenal cavernal (or cavernous) hemangioma -which is the most frequent type of AH- is a well-demarcated lesion [4,7]:

- Histologically made of large linear cavities that contain erythrocytes and thrombi, bordered by single-layer vascular endothelium
- Eroding and pressuring neighboring structures
- With calcifications and other degenerative modifications (thrombi, hemorrhage and necrosis), which are not specific of this type of neoplasm.
- Positive for CD31, C34 and ERG on immunohistochemistry.

Usually seen in 50–70 year-old patients, Adrenal hemangiomas are, most of the time, unilateral lesions. They tend to affect more women than men (sex-ratio 3:2 to 2:1) [[2], [3], [4]].

Incidental discovery is the most frequent discovery circumstance of AH. Symptomatic or clinically palpable AH, are uncommon, but reported, situations. Pain, palpable abdominal/lumbar mass, arterial hypertension, urinary/gastro-intestinal symptoms, anemia and deterioration of general condition can reveal these tumors [4]. Exceptionally, a case of cardiovascular collapse with retroperitoneal hemorrhage due to adrenal hemangioma has been reported [13]. In our case, lumbar chronic pain was the revealing symptom, which is not usual.

AH can be associated with malignant lung neoplasms (small-cell lung cancer), gynecologic or colic tumors (familial adenomatous polyposis). AH can also be associated with neonatal syndromes such as Klippel-Trenaunay or Sturge-Weber syndromes [14,15].

Adrenal hemangiomas are known to be non-functional tumors. Functional evaluation of these tumors is typically normal. However, six cases of secreting AH (aldosterone and catecholamines) have been reported in the literature [5].

CT scan and MRI characteristics of AH are suggestive of its nature, but not always specific.

The classic appearance of AH on imaging associates [5,[16], [17], [18], [19]]:

- A highly dense peripheral edge on CT scan
- A peripheral patchy enhancement
- Spontaneously dense spots revealing calcification areas
- Hypointense lesion on T1-weighted images
- Hyperintense lesion on T2-weighted images
- No signal decrease in out-of-phase (OOP) MRI sequence

AH can mimic other benign (adenoma) or malignant (adrenocortical carcinoma) lesions on imaging. 18 FDG – PET scan is helpful when atypical characteristics are found on conventional cross-sectional imaging techniques. It shows a low-uptake lesion. Neither MRI and PET scan were judged futile in our case.

Adrenal hemangiomas bear the risk of spontaneous hemorrhage and abdominal compression syndrome as they increase in size [20,21]. The causality link between lumbar chronic pain and adrenal hemangioma seems plausible. It could be explained by the mechanical mass effect of the adrenal neoplasm on neighboring structures.

Surgical intervention remains the gold standard of treatment [22]. Resection of adrenal neoplasms, including adrenal hemangiomas, is preconized in these situations, according to European and American recommendations [23].

- The mass shows clinical or radiological features of malignancy: DGC, neoplasm's size (> 4–5 cm), spontaneous density on CT-scan (> 10 HU), absolute/relative wash-out (<60 and 40 %, respectively) on CT-scan or MRI, no signal decrease on out-of-phase MRI sequence, and hypermetabolism on PET-scan.
- The mass is complicated and/or biologically functional

In the other situations, the decision is debatable: a conservative treatment is defensible. A radical treatment was chosen for our patient given the fact the neoplasm was symptomatic and possibly malignant.

Surgical resection of incidentalomas is useful for diagnostic and therapeutic purposes:

- It confirms the histological type of the lesion. Indeed, definitive diagnosis of the adrenal mass is made by histopathologic study of the adrenalectomy specimen.
- It prevents the risk of spontaneous hemorrhage of adrenal hemangiomas, and helps achieve symptom relief by eliminating the mass effect of the lesion on adjacent abdominal structures.

Laparoscopic approach is preferred, if technically feasible, as it is associated with a quicker post- operative recovery, compared to open approach [24]. However, open approach, −through an anterior (subcostal or midline incision), posterior or thoraco-abdominal approach [15,25]- is indicated when the tumor is large (above 6 cm) or suspected to be locally invasive.

A multidisciplinary discussion and literature analysis of cases of adrenal hemangioma seems to be necessary due to the rarity of the disease, its challenging preoperative diagnosis, its uncertain malignant potential, its evolutionary risks and the scarcity of literature on the subject.

## 4 Conclusion

Adrenal cavernous hemangioma is a rare benign vascular tumor, often discovered on adrenalectomy specimen. Surgery and conservative management must consider the benefit-risk balance, emphasizing a multidisciplinary approach and patient involvement.

## Patient consent

Patient gave informed consent before publication of the case report and accompanying illustrations. A copy of the written consent is available for review by the Editor-in-Chief of this journal on request.

## Provenance and peer review

Not commissioned, externally peer-reviwed.

## Ethical approval

Ethical approval is not required for case reports or case series deemed not to constitute research at my institution. The case report is not containing any personal information.

## Funding

State N/A.

## Author contribution

All authors contributed to the final approval of the version to be published. All authors read a nd approved the final manuscript.

ktari Ktari kamel: study concept

Jelassi Mohamed Amine and Wadii Hamdouni^1^ data collection and writing

Seifeddine Ben Hamouda ^2^ and Manel Njim^2^, data analysis or interpretation of histological section

Fedia Boubaker^3^, data analysis or interpretation of endocrinological exploration

Jamel Saad ⁴ data analysis or interpretation of imagery

## Guarantor

Ktari Kamel

## Research registration number

1. Name of the registry:N/A

2. Unique identifying number or registration ID: N/A

3. Hyperlink to your specific registration (must be publicly accessible and will be checked): N/A

## Conflict of interest statement

State N/A.
